# Schwerverletztenversorgung durch Notärzte aus unterschiedlichen Fachgebieten: Eine retrospektive, multizentrische Untersuchung aus Daten der ADAC Luftrettung und des deutschen TraumaRegister DGU®

**DOI:** 10.1007/s00113-021-01094-4

**Published:** 2021-10-26

**Authors:** Michael Gäßler, Matthias Ruppert, Rolf Lefering, Bertil Bouillon, Arasch Wafaisade, TraumaRegister DGU

**Affiliations:** 1 Fachbereich Medizin, ADAC Luftrettung, Hansastr. 19, 80686 München, Deutschland; 2 Institut für Forschung in der Operativen Medizin (IFOM), Universität Witten/Herdecke, Ostmerheimer Str. 200, 51109 Köln, Deutschland; 3 Klinik für Orthopädie, Unfallchirurgie und Sporttraumatologie, Lehrstuhl der Universität Witten/Herdecke, Klinikum Merheim – Kliniken der Stadt Köln, Ostmerheimerstr. 200, 51109 Köln, Deutschland; 4 Sektion Notfall‑, Intensivmedizin und Schwerverletztenversorgung (Sektion NIS), Deutsche Gesellschaft für Unfallchirurgie (DGU), Berlin, Deutschland

**Keywords:** Polytrauma, Notfallmedizin, Fachrichtung, Intubation, Rettungsdienst, Polytrauma, Emergency medicine, Medical specialty, Intubation, Emergency service

## Abstract

**Hintergrund und Ziel:**

Die S3-Leitlinie Polytrauma/Schwerverletzten-Behandlung stellt den definierten Rahmen für eine leitliniengerechte Erstversorgung des schwer verletzten Patienten dar. Mutmaßlich werden diagnostische und therapeutische Entscheidungsfindungen in der Präklinik durch die klinische Expertise aus dem Fachgebiet des Notarztes mitbeeinflusst.

**Material und Methoden:**

Retrospektive, multizentrische Studie aus Daten der ADAC (Allgemeiner Deutscher Automobil-Club e. V.) Luftrettung und des TraumaRegister der Deutschen Gesellschaft für Unfallchirurgie®. Im Untersuchungszeitraum von 2011 bis 2015 konnten 11.019 schwer verletzte Patienten eingeschlossen und der Versorgung durch Notärzte aus den Fachgebieten Anästhesie (ANÄ), innere Medizin (INN) und Chirurgie (CHIR) zugeordnet werden.

**Ergebnisse:**

Durch ANÄ wurden 81,9 %, durch INN 7,6 % und durch CHIR 10,5 % versorgt. Präklinisch wurden 40,5 % der Patienten intubiert (ANÄ 43,0 %, INN 31,2 %, CHIR 28,3 %; *p* < 0,001), 5,5 % haben eine Thoraxdrainage (ANÄ 5,9 %, INN 4,2 %, CHIR 2,8 %; *p* = 0,004) und 10,8 % eine Katecholamintherapie erhalten (ANÄ 11,3 %, INN 8,3 %, CHIR 8,3 %; *p* = 0,022). Bewusstlose Patienten wurden in 96,0 % intubiert (ANÄ 96,1 %, INN 97,7 %, CHIR 93,9 %; *p* = 0,205). Die Mortalität wurde nicht durch die fachliche Herkunft des Notarztes beeinflusst.

**Diskussion:**

In diesem Kollektiv aus dem Bereich der Luftrettung zeigten sich in den Gruppen Unterschiede bei der Indikationsstellung zu invasiven Maßnahmen, die möglicherweise durch die jeweilige klinische Expertise bedingt sind. Am Beispiel der Intubation konnte gezeigt werden, dass Leitlinienempfehlungen in hohem Maße – unabhängig von der Fachgebietszugehörigkeit des Notarztes – umgesetzt werden.

## Abstract (en)

**Background and objective:**

The level 3 guidelines on treatment of patients with severe/multiple injuries provide a defined framework for an appropriate treatment of these patients. It is presumed that prehospital diagnostic and therapeutic decisions are affected by the clinical expertise and the medical disciplines of the emergency physicians.

**Methods:**

Retrospective, multicenter study based on data from the ADAC Air Recue Service and the TraumaRegister DGU®. In the study period 2011–2015, a total of 11,019 seriously injured patients were included. They were treated by emergency physicians from the following disciplines: anesthesiology (ANÄ), internal medicine (INN) and surgery (CHIR).

**Results:**

Of the patients 81.9% were treated by ANÄ, 7.6% by INN and 10.5% by CHIR. Preclinically, 40.5% of patients were intubated (ANÄ 43.0%, INN 31.2%, CHIR 28.3%; *p* < 0.001), 5.5% received pleural decompression (ANÄ 5.9%, INN 4.2%, CHIR 2.8%; *p* = 0.004),and 10.8% were treated with catecholamines (ANÄ 11.3%, INN 8.3%, CHIR 8.3%; *p* = 0.022). Unconscious patients were intubated in 96.0% (ANÄ 96.1%, INN 97.7%, CHIR 93.9%; *p* = 0.205). The mortality was not influenced by the medical specialty of the emergency physician.

**Conclusion:**

In this air rescue cohort differences in indications for invasive procedures were observed between the groups. This may be caused by their clinical background. Using the example of intubation, it has been shown that guideline recommendations were closely followed irrespective of the medical specialty of the emergency physician.

## Hintergrund und Fragestellung

In der Bundesrepublik Deutschland besteht ein flächendeckendes, notarztgestütztes Rettungsdienstsystem [24]. Die Notärzte rekrutieren sich überwiegend aus den 3 großen akutmedizinischen Fachgebieten Anästhesie (ANÄ), innere Medizin (INN) und Chirurgie (CHIR) [9, 15, 22, 30]. Die beiden wesentlichen deutschen Luftrettungsorganisationen (Allgemeiner Deutscher Automobil-Club e. V./ADAC Luftrettung, Deutsche Rettungsflugwacht/DRF Luftrettung) führen bereits seit über 2 Jahrzehnten eine medizinische Qualitätssicherung mit eigenen Registern durch [8, 26]; landesweite Auswertungen des rettungsdienstlichen Einsatzgeschehens sind inzwischen auch in den Bundesländern Baden-Württemberg und Bayern etabliert [16, 20, 21].

Präklinische Versorgungsforschung scheitert häufig am fehlenden Zugang zu Verlaufs- und Outcome-Parametern aus der weiteren klinischen Versorgung. Es besteht der dringende Bedarf für sektorenübergreifende Ansätze, welche bisher nur für das TraumaRegister DGU® der Deutschen Gesellschaft für Unfallchirurgie (TR-DGU), das deutsche Reanimationsregister sowie für singuläre Fragestellungen umgesetzt sind [6, 7, 19, 29]. Ergebnisse aus solchen Datenpools erlauben – neben der Prozessqualität – auch die Bewertung von einzelnen Aspekten der (präklinischen) Ergebnisqualität.

Die S3-Leitlinie Polytrauma‑/Schwerverletzten-Behandlung gibt dem Notarzt einen definierten Rahmen für eine erkenntnis- bzw. konsensbasierte Erstversorgung des schwer verletzten Patienten [5]. Notärztliche Therapiequalität geht jedoch über eine rein leitlinien- und algorithmenbasierte Patientenbehandlung hinaus. Die klinische Erfahrung aus dem jeweiligen Fachgebiet wird, insbesondere bei komplexen differenzialdiagnostischen Überlegungen und schwierigen therapeutischen Indikationsstellungen, die präklinische Entscheidungsfindung mitbeeinflussen. Durch die Datenfusion von ADAC Luftrettung und TR-DGU ist es erstmals möglich, notärztliche Prozess- und Therapiequalität in diesem Umfang und unter dieser Fragestellung zu untersuchen.

## Studiendesign und Untersuchungsmethoden

**Figure 1:**
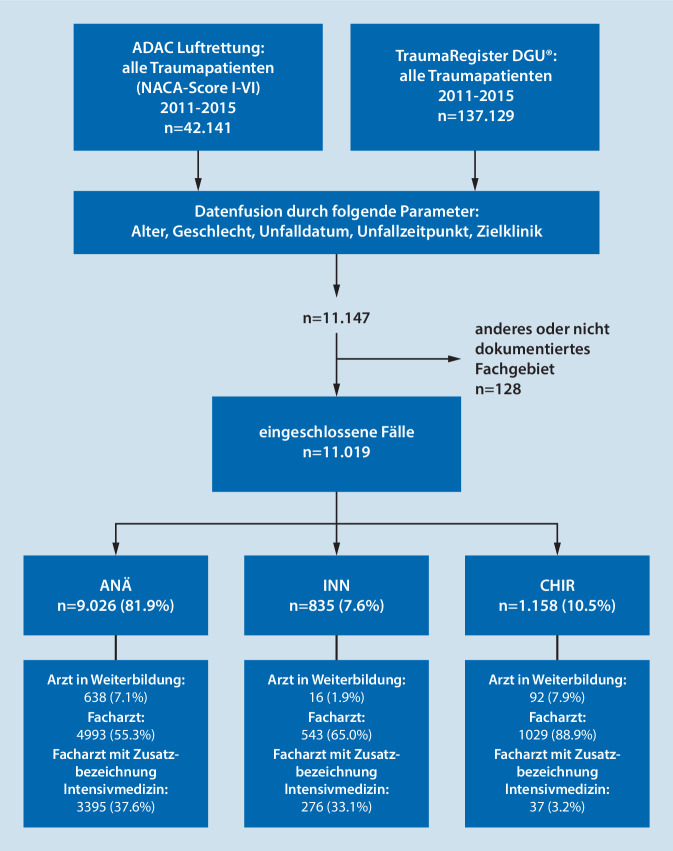

Grundlage dieser retrospektiven, multizentrischen Studie sind 2 große akutmedizinische Register. Für den Untersuchungszeitraum vom 01.01.2011 bis zum 31.12.2015 wurde eine Datenfusion zwischen allen traumatologischen Diagnosen der elektronischen Einsatzdatenbank der ADAC Luftrettung (National Advisory Committee for Aeronautics [NACA] Scores I–VI) und dem TR-DGU durchgeführt. Über die Parameter Alter, Geschlecht, Unfalldatum, Unfallzeitpunkt und Zielklinik wurden die Patienten, welche in beiden Registern erfasst sind, zusammengeführt. Diese pseudonymisierte Datenfusion konnte bereits im Rahmen anderer Studien erfolgreich angewandt werden [6, 29]. Insgesamt konnten für 11.147 Fälle Datensätze aus beiden Registern zusammengeführt werden („matching“) und davon 11.019 Patientenversorgungen einem der 3 notärztlichen Fachgebiete ANÄ, INN oder CHIR zugeordnet werden (Abb. 1).

Die vorliegende Arbeit steht in Übereinstimmung mit der Publikationsrichtlinie des TR-DGU und ist registriert unter der ID 2016-009. Eine Genehmigung zur Nutzung der pseudonymen Daten vonseiten der ADAC Luftrettung ist ebenfalls erteilt. Es liegt ein positives Votum der Ethik-Kommission der Universität Witten/Herdecke vor (Antrag-Nr. 158/2019).

### Datenerhebung und Zuordnung zum jeweiligen Register

Der Datensatz der ADAC Luftrettung wird routinemäßig innerhalb jedes Einsatzes erhoben. Die Dokumentation erfolgt papiergestützt auf dem Notarzteinsatzprotokoll und wird anschließend digital in die elektronische Einsatzdatenbank überführt. Hier kommt das Konzept eines „digital pen/digital paper system“ zur Anwendung. Es erfolgt zudem eine Verifikation der Daten bei der Übertragung in die Einsatzdatenbank durch den Notarzt [11]. Grundlage der medizinischen Einsatzdokumentation ist der Minimale Notarztdatensatz 2 (MIND2) [23].

Das TR-DGU ermöglicht seit 1993 eine pseudonymisierte und standardisierte Dokumentation von Schwerverletzten in einer multizentrischen Datenbank. Die Daten werden prospektiv in 4 aufeinanderfolgenden Phasen gesammelt: A) präklinische Phase, B) Schockraum‑/Operationsphase, C) Intensivstation und D) Entlassung. Der Erhebungsbogen des TraumaRegister DGU® liegt in 2 Versionen vor. Der Standardbogen verlangt die Eingabe von ca. 100 Parametern. Daneben liegt eine gekürzte Version mit ca. 40 Parametern vor, der sog. QM-Bogen.

Im TR-DGU sind alle Patienten dokumentiert, welche in teilnehmenden Kliniken über den Schockraum aufgenommen wurden und eine anschließende Intensivtherapie benötigten oder noch vor Aufnahme auf die Intensivstation verstarben. Für alle Patienten aus dem Datensatz der ADAC Luftrettung, welche diese Einschlusskriterien nicht erfüllten, konnte dementsprechend kein korrespondierender Datensatz gefunden werden.

Nach Zusammenführung der Daten ist ein kumulierter Datensatz aus beiden Registern mit definierten Parametern für die Präklinik (PK) und den Schockraum (SR) entstanden. Die Daten der ADAC Luftrettung dienten in erster Linie der Zuordnung zum jeweiligen notärztlichen Fachgebiet. Darüber hinaus wurden die präklinischen Interventionen intraossärer Zugang, Reposition und eingesetzte Analgetika (Opioide, Ketamin, periphere Analgetika) aus dem ADAC-Datensatz extrahiert.

Alle anderen Interventionen, alle Vital- und Zeitparameter sowie die Outcome-Daten sind dem TR-DGU entnommen. Folgende Variablen sind für alle Patienten erfasst: Alter, Geschlecht, Unfallmechanismus, Injury Severity Score (ISS), Abbreviated Injury Scale (AIS), systolischer Blutdruck (SBP) (PK + SR), Atemfrequenz (AF) (PK), Glasgow Coma Scale (GCS) (PK), Intubation (PK), Reanimation (PK), Beatmungstage, Intensivtage, Krankenhaustage, Mortalität.

Diese Parameter sind nur im Standardbogen abgebildet und damit nur für einen Teil der Patienten verfügbar: Sauerstoffsättigung (S_p_O_2_) (PK), GCS (SR), Intubation (SR), Reanimation (SR), Katecholamine (PK + SR), Thoraxdrainage (PK + SR), Versorgungszeit (Ankunft des Notarztes an der Einsatzstelle bis Transportbeginn), Schockraumzeit.

### RISC-II-Score

Der Revised Injury Severity Classification (RISC)-Score, Version II, wurde an 30.000 Patienten aus dem TR-DGU entwickelt und anschließend validiert [18]. Er kombiniert 13 Angaben zum Patienten bis kurz nach der Aufnahme im Krankenhaus (Alter, schwerste und zweitschwerste Verletzung nach AIS; Kopfverletzung; Pupillenweite und -reaktion; GCS, ASA-Klassifikation, Unfallmechanismus; Geschlecht; Blutdruck; Basenabweichung; International Normalized Ratio (INR); Hämoglobin(Hb)-Werte; Reanimation) und erstellt daraus eine individuelle Mortalitätsprognose. Für Gruppen von Patienten wird der Mittelwert der RISC-II-Prognosen dann mit der tatsächlich beobachteten Mortalitätsrate verglichen.

### Statistik

Die Zusammenführung der beiden Datensätze und die statistische Auswertung erfolgten in SPSS® (Version 22.0, IBM, Armonk/NY, USA). Unterschiede zwischen den 3 Gruppen wurden mithilfe des Chi-Quadrat-Testes (kategoriale Daten) und des Kruskal-Wallis-Testes (metrische Daten) statistisch geprüft. Die Darstellung erfolgte mit Mittelwert (MW) und Standardabweichung (SD) für metrische Daten; ergänzt durch den Median bei schief verteilten Daten. Kategoriale Daten sind in Prozent angegeben. Ein *p*-Wert < 0,05 wurde als signifikant definiert. Für ausgewählte Ergebnisse wurden 95 %-Konfidenzintervalle (95 %-KI) bestimmt. Dabei ist zu bedenken, dass die Studie primär einen deskriptiven Charakter hat und durch multiple Vergleiche sowie durch die große Fallzahl eine formale statistische Signifikanz nicht immer klinisch relevant ist.

Der Einfluss der Fachrichtung auf die Krankenhausmortalität wurde multivariat mithilfe einer logistischen Regression untersucht. Neben der Fachrichtung wurden folgende weitere Prädiktoren als unabhängige Variablen im Modell mitbetrachtet: Bewusstlosigkeit, Injury Severity Score (ISS), relevante Verletzungen (AIS ≥ 3) von Kopf, Thorax, Abdomen und Extremitäten; eine präklinische Reanimation, Geschlecht, Alter (in 5 Gruppen), Unfallmechanismus und die Versorgungsstufe des aufnehmenden Krankenhauses. Die Ergebnisse werden als „odds ratios“ mit 95 %-KI dargestellt.

## Ergebnisse

In diese Studie konnten 11.019 Patienten eingeschlossen werden. Die Verteilung auf die Fachgebiete sowie die Qualifikation der Notärzte ist Abb. 1 zu entnehmen.

Demografische Daten, Patientencharakteristika und Verletzungsschwere sind Tab. 1 zu entnehmen. Das durchschnittliche Alter ist 46 Jahre, 73 % sind männlichen Geschlechts, der ISS liegt bei 19 Punkten. Der ISS ist in CHIR niedriger als in den beiden anderen Gruppen (*p* < 0,001). In ANÄ sind Schädel-Hirn-Traumata am häufigsten (*p* < 0,001).

**Table 1:** 

	ANÄ *n* = 9026	INN *n* = 835	CHIR *n* = 1158	*p*-Wert
Alter (Jahre)^a^	46 ± 21; 46	46 ± 21; 47	45 ± 20; 46	0,38
Männliches Geschlecht (*n*, %)	6630 (73,5 %)	600 (71,9)	859 (74,2)	0,50
Unfallmechanismus, stumpf (*n*, %)	8068 (95,8)	760 (96,0)	1041 (95,7)	0,95
*Verletzungsschwere*				
ISS (Punkte)^a^	19 ± 13; 17	19 ± 13; 17	17 ± 13; 14	<0,001
AIS_HEAD_ ≥ 3 (*n*, %)	2933 (32,5)	246 (29,5)	303 (26,2)	<0,001
AIS_THORAX_ ≥ 3 (*n*, %)	3646 (40,4)	346 (41,4)	442 (38,2)	0,27
AIS_ABDOMEN_ ≥ 3 (*n*, %)	981 (10,9)	90 (10,8)	96 (8,3)	0,027
AIS_EXTREMITIES_ ≥ 3 (*n*, %)	2476 (27,4)	233 (27,9)	317 (27,4)	0,96
*Vitalparameter bei Ankunft des Notarztes*				
AF^a^ (1/min)	16 ± 6; 15	15 ± 6; 14	15 ± 5; 14	0,001
S_p_O_2_ (%)^a^	94 ± 11; 96	94 ± 8; 96	94 ± 10; 96	0,99
SBP (mm Hg)^a^	129 ± 32; 130	129 ± 33; 130	129 ± 30; 130	0,84
SBP ≤ 90 mm Hg (*n*, %)	916 (11,4)	84 (11,0)	98 (9,6)	0,24
GCS^a^	12 ± 4; 15	13 ± 4; 15	13 ± 4; 15	<0,001
GCS ≤ 8 (*n*, %)	1.757 (21,5)	134 (17,0)	182 (16,6)	<0,001

*ISS* Injury Severity Score, *AIS* Abbreviated Injury Scale, S_p_O_2_ Sauerstoffsättigung, *SBP* systolischer Blutdruck, *GCS* Glasgow Coma Scale, *AF* Atemfrequenz

^a^Stetige Messwerte dargestellt als Mittelwert ± Standardabweichung; Median

### Intubation

**Figure 2:**
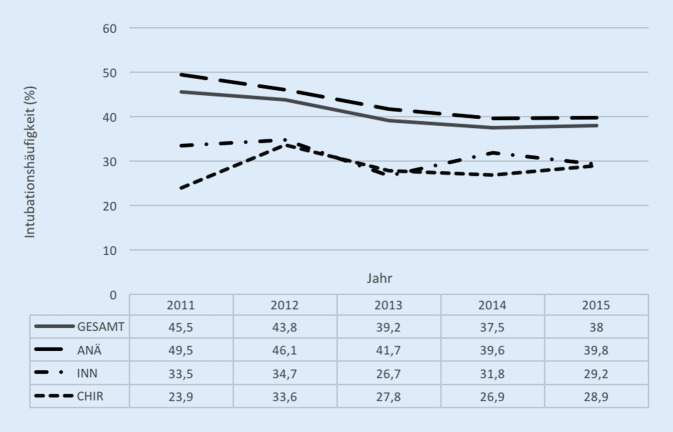

Die Intubationshäufigkeit in den einzelnen Gruppen zeigt Tab. 2. Präklinische Intubationen waren über den Beobachtungszeitraum in der Gesamtgruppe rückläufig, wobei sich besonders in ANÄ ein Rückgang und in CHIR eine Zunahme zeigen (Abb. 2).

**Table 2:** 

	ANÄ	INN	CHIR	*p*-Wert
**Interventionen, Präklinik**				
Intubation (*n*, %)	3766 (43,0)	254 (31,2)	320 (28,3)	<0,001
Thoraxdrainage (*n*, %)	284 (5,9)	19 (4,2)	15 (2,8)	0,004
Reanimation (*n*, %)	120 (2,5)	11 (2,4)	18 (3,3)	0,050
Katecholamine (*n*, %)	542 (11,3)	38 (8,3)	45 (8,3)	0,022
Volumengabe^a^ (ml)	970 ± 633; 1000	899 ± 610; 600	847 ± 533; 500	<0,001
Intraossärer Zugang (*n*, %)	148 (1,6 %)	16 (1,9 %)	13 (1,1 %)	0,32
Reposition (*n*, %)	1082 (12,0 %)	103 (12,3 %)	174 (15,0 %)	0,013
Analgosedierung (*n*, %)	3591 (74,6 %)	343 (75,1 %)	395 (72,7 %)	0,61
*Opioide (n, %)*	*6596 (73,1* *%)*	*584 (69,9* *%)*	*738 (63,7* *%)*	*<0,0001*
*Ketamin (n, %)*	*1953 (21,1* *%)*	*131 (15,7* *%)*	*229 (19,8* *%)*	*<0,0001*
*Periphere Analgetika (n, %)*	*212 (2,3* *%)*	*14 (1,7* *%)*	*35 (3,0* *%)*	*0,14*
**Interventionen, Schockraum**				
Intubation (*n*, %)	559 (11,4)	35 (7,5)	73 (13,5)	0,010
Thoraxdrainage (*n*, %)	561 (11,4)	52 (11,2)	60 (11,1)	0,96
Reanimation (*n*, %)	130 (2,7)	7 (1,5)	26 (4,8)	0,003
Katecholamine (*n*, %)	1028 (21,0)	79 (17,0)	84 (15,5)	0,002
**Zeitintervalle**				
Präklinische Versorgungszeit^a^	31 ± 17; 27	29 ± 16; 25	25 ± 14; 22	<0,001
Schockraumzeit^a^	59 ± 38; 50	51 ± 37; 38	50 ± 34; 40	<0,001
Beatmungstage^a^	3,7 ± 8,3; 0	3,3 ± 8,0; 0	3,4 ± 9,0; 0	<0,001
Intensivtage^a^	7,1 ± 11,0; 2	6,5 ± 10,7; 2	6,4 ± 11,2; 2	<0,001
Krankenhaustage^a^	18,5 ± 20,8; 13	17,0 ± 16,8; 12	19,3 ± 23,0; 12	0,413
**Mortalität**				
Krankenhausmortalität (*n*, %)^b^	882 (10,4)	62 (7,9)	87 (8,0)	0,008
95 %-KI der Mortalität^b^	9,7–11,0	6,0–9,8	6,4–9,8	–
RISC-II-Prognose für Mortalität (%)^b^	10,7	9,5	8,6	<0,001
„Standard mortality ratio“ (SMR)	0,97	0,83	0,93	–
95 %-KI der SMR	0,91–1,03	0,63–1,03	0,74–1,14	–

^a^Stetige Messwerte dargestellt als Mittelwert ± Standardabweichung; Median

^b^Nur ausbehandelte Patienten mit Prognose; ohne Zu- und Weiterverlegte (*n* = 10.539)

**Figure 3:**
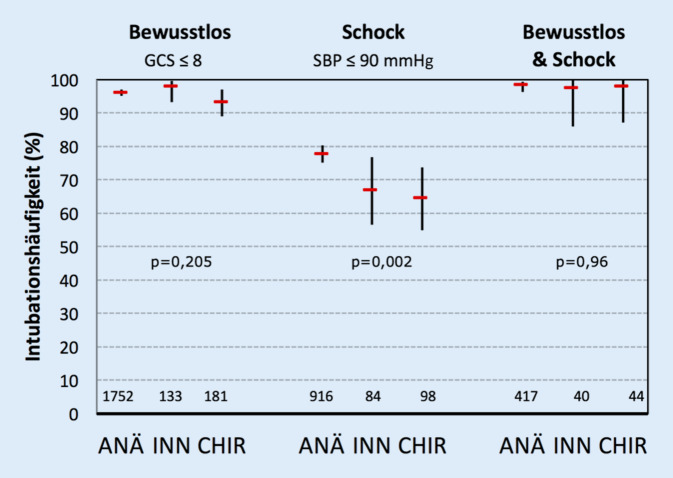

Bewusstlose Patienten (GCS ≤ 8) wurden im Gesamtkollektiv in 96,0 % (95 %-KI 95,1–96,9), Patienten im Schock (SBP ≤ 90 mm Hg) in 75,7 % (95 %-KI 70,6–81,0), bewusstlose und kreislaufinstabile Patienten in 98,0 % (95 %-KI 96,3–99,0) präklinisch intubiert. Die Ergebnisse für die einzelnen Fachgebiete zeigt Abb. 3.

Ohne formale Intubationsindikation anhand der initial erhobenen Vitalparameter (AF, S_p_O_2_, SBP oder GCS) wurden in ANÄ 26,4 %, in INN 16,6 % und in CHIR 14,1 % intubiert (*p* < 0,001).

Bei der Übergabe im Schockraum war folgender Anteil an Patienten bewusstlos (GCS ≤ 8) und nicht intubiert: ANÄ 2,6 %, INN 3,9 %, CHIR 9,8 % (*p* < 0,001) sowie im Schock (SBP ≤ 90 mm Hg) und nicht intubiert: ANÄ 15,7 %, INN 21,7 %, CHIR 19,5 % (*p* = 0,32).

### Häufigkeit weiterer Interventionen

Die Durchführung weiterer Interventionen in der Präklinik und im Schockraum ist Tab. 2 zu entnehmen.

### Zeitparameter und Mortalität

Die Versorgungszeit am Unfallort, die Dauer der Schockraumversorgung, Beatmungs- und Intensivtage sowie die Krankenhausmortalität mit der RISC-II-Prognose sind in Tab. 2 dargestellt.

Beim Vergleich von Prognose (RISC II) und Mortalität zeigen sich keine relevanten Unterschiede; in allen drei Gruppen liegt die Prognose innerhalb des 95 %-KI der Sterblichkeit. In einer multivariablen Analyse mit der Krankenhaussterblichkeit als abhängige Variable zeigt sich kein Einfluss des fachlichen Hintergrundes des Notarztes auf die Mortalität beim Schwerverletzten. Im Vergleich zu ANÄ (Referenz) ergibt sich eine Odds Ratio für INN von 0,77 (95 %-KI 0,53–1,12) und für CHIR von 0,86 (95 %-KI 0,61–1,22).

## Diskussion

In einem primär deskriptiven Ansatz stellt die vorliegende Arbeit Aspekte der präklinischen Prozess- und Therapiequalität im Rahmen der Schwerverletztenversorgung unter Berücksichtigung der fachlichen Herkunft der Notärzte im Luftrettungsdienst dar. Am Beispiel der Intubation konnte gezeigt werden, dass Leitlinienempfehlungen in hohem Maße – unabhängig von der Fachgebietszugehörigkeit – umgesetzt werden. Anhand einzelner Interventionen können Unterschiede in der notärztlichen Entscheidungsfindung bzw. Indikationsstellung zwischen den Fachgebieten aufgezeigt werden, die zumindest auf das Überleben keinen statistisch nachweisbaren Einfluss haben.

Zum Einfluss des fachlichen Hintergrundes des Notarztes gibt es außerhalb des Bereiches Notfallnarkose und Atemwegsmanagement kaum Daten [2, 22, 30]. Notärzte in der Luftrettung rekrutieren sich überwiegend aus dem Fachgebiet Anästhesie (82 % der Patientenversorgungen in der vorliegenden Studie). Im Vergleich dazu wurden in Baden-Württemberg in den Jahren 2015–2017 53 % der Notarzteinsätze durch Anästhesisten, 19 % durch Internisten und 8 % durch Chirurgen durchgeführt [22]. Für die Tätigkeit bei der ADAC Luftrettung werden eine Facharztanerkennung oder zumindest die Facharztreife sowie umfangreiche Erfahrungen im bodengebundenen Rettungsdienst vorausgesetzt. Im Luftrettungsdienst werden signifikant häufiger Schwerverletzte versorgt [9], was – unabhängig vom Fachgebiet – zu einem hohen „case load“ in der Traumaversorgung führt.

### Präklinische Interventionen

Für die im vorliegenden Kollektiv aufgezeigten Unterschiede in Bezug auf die Durchführung präklinischer Interventionen gibt es mehrere Interpretationsmöglichkeiten.

In ANÄ werden signifikant mehr Patienten intubiert als in den Vergleichsgruppen. Dies erklärt sich z. T. durch einen höheren Anteil an Schädel-Hirn-Traumata. Darüber hinaus scheint aber der fachliche Hintergrund die Intubationsindikation, besonders außerhalb der formalen Intubationskriterien, zu beeinflussen.

Einerseits erscheint es denkbar, dass eine hohe Expertise in der Durchführung einer therapeutischen Maßnahme per se zu einer „liberaleren“ Indikationsstellung führt. Diesen möglichen Zusammenhang postulieren bereits Breckwoldt et al. am Beispiel der Intubation [2]. Ähnlich könnte die höhere Inzidenz von Repositionen in CHIR erklärt werden.

Andererseits könnte eine große individuelle Erfahrung mit dem frühklinischen Verlauf von Traumapatienten auch zu einer „vorausschauenden“ Indikationsstellung führen, bei der eine Entwicklung der Vitalfunktionen präjudiziert wird. Hierfür spräche der hohe Anteil von Patienten in ANÄ, die präklinisch intubiert werden, ohne dass sich eine formale Intubationsindikation ableiten lässt – aber gleichzeitig dem geringsten Anteil von Patienten in dieser Gruppe, die zum Zeitpunkt der Schockraumaufnahme bewusstlos und nicht intubiert sind.

Insgesamt ist die Häufigkeit präklinischer Intubationen bei Traumapatienten über die letzten Jahre rückläufig [1, 3, 4]. Die vorliegenden Ergebnisse zeigen jedoch unterschiedliche Entwicklungen: in ANÄ und INN wurden weniger, in CHIR mehr Patienten intubiert. Diese Annäherung der Intubationsindikationen kann als Ausdruck des hohen Durchdringungsgrades der S3-Leitlinie Polytrauma interpretiert werden.

Bei ähnlicher Inzidenz schwerer Thoraxtraumata in den Gruppen ist die Häufigkeit der Anlage von Thoraxdrainagen in ANÄ am größten. Mutmaßlich mündet die höhere Anzahl von Intubationen konsekutiv auch in einem höheren Bedarf, (Spannungs‑)Pneumothoraces unter Beatmungsbedingungen zu dekomprimieren. Ebenso werden Katecholamine in ANÄ am häufigsten indiziert; möglicherweise ist auch dies durch die höhere Anzahl präklinischer Notfallnarkosen und eine konsekutive Hypotension bedingt, die wiederum bei einem hohen Anteil an Schädel-Hirn-Traumata in dieser Gruppe konsequent therapiert wird. Eine andere Interpretationsmöglichkeit ist, dass eine stärkere fachgebietsspezifische Fokussierung auf „Vitalparameter im Normbereich“ i. Allg. sowie der routinierte Umgang mit Katecholaminen im klinischen Alltag zu einer großzügigeren Anwendung führen.

Intraossäre Zugänge werden in allen Gruppen selten eingesetzt. Die Inzidenz lag etwas über den Beobachtungen vorhergehender präklinischer Untersuchungen (0,3–0,9 %), welche jedoch ebenfalls eine häufigere Anwendung bei Traumapatienten zeigten [12, 14, 25]. Präklinische Analgesie wird von stark wirksamen Opioiden dominiert [13], so auch in allen 3 Gruppen in dieser Studie. In CHIR wurde Ketamin – im Vergleich zu Opioiden – relativ häufiger eingesetzt als in ANÄ und INN. Eine aktuelle Metaanalyse konnte keine Evidenz für die Überlegenheit einer der 3 Substanzen Fentanyl, Morphin oder Ketamin beim Traumapatienten zeigen [10].

Außerhalb der definierten Indikationen beeinflussen wohl die (prä-)klinische Erfahrung sowie der fachliche Hintergrund die Entscheidungsfindung bezüglich Indikation und Zeitpunkt einer invasiven Maßnahme.

Unter den Bedingungen des luftgestützten Patiententransportes müssen im Sinne einer optimalen Patientensicherheit die speziellen Transportmodalitäten eine besondere Beachtung finden. In dieser Studie lag die Intubationshäufigkeit in allen 3 Facharztgruppen über dem Gesamtdurchschnitt des TR-DGU [1, 3, 4]. Bereits eine Untersuchung aus den Jahren 2005–2011 zeigte, dass im luftgebundenen Notarztdienst – bei vergleichbaren demografischen Daten und Verletzungsmustern – mehr Maßnahmen durchgeführt wurden als im bodengebundenen Notarztdienst (z. B. Intubation 62,9 % vs. 33,9 %; Thoraxdrainagen 9,4 % vs. 2,9 %) [27]. Potenzielle Gründe hierfür mögen eine hohe Leitlinienadhärenz und Expertise der Notärzte im Luftrettungsdienst sowie die oft längeren Transportwege und die eingeschränkten Interventionsmöglichkeiten im Hubschrauber sein. Unter Berücksichtigung der vorliegenden Ergebnisse wirkt sich möglicherweise aber auch der hohe Anteil an Anästhesisten in der Luftrettung auf die Inzidenz invasiver Maßnahmen aus.

### Umsetzung der S3-Leitlinie Polytrauma

Die S3-Leitlinie Polytrauma/Schwerverletzten-Behandlung enthält 70 Empfehlungen für den Bereich der Präklinik [5]. Bei den korrespondierenden Indikationsstellungen handelt es sich überwiegend um Entscheidungsfindungsprozesse, welche nur in sehr begrenztem Umfang mit einem elektronischen Datensatz überprüft werden können. Für die präklinische Intubation kann jedoch auf Grundlage der dokumentierten Vitalparameter eine leitliniengerechte Intubationsindikation weitgehend nachvollzogen werden, auch unter Berücksichtigung der Vitalparameter bei Übergabe im Schockraum.

Leitlinienempfehlungen bezüglich Strukturqualität sind in der Luftrettung weitgehend umgesetzt. Für die Empfehlung, kein Etomidat als Einleitungshypnotikum zu verwenden, konnte bereits eine hohe Leitlinienadhärenz gezeigt werden [6, 28].

Die Aspekte rund um die präklinische Intubation schwer verletzter Patienten zeigen zudem, dass eine reine Betrachtung des Erfüllungsgrades von Leitlinienempfehlungen kein Abbild der tatsächlichen, individuellen Therapiequalität darstellen kann. So wird offensichtlich in diesem Kollektiv bei der Indiktionsstellung zur Intubation des bewusstseinsgetrübten Patienten auch eine potenzielle Dynamik während des präklinischen Verlaufs berücksichtigt, da zum Zeitpunkt der Übergabe im Schockraum ebenfalls (noch) ein hohes Maß an Leitlinienerfüllung besteht. In ANÄ und INN wird diese Strategie wohl etwas stringenter als in CHIR verfolgt, wo möglicherweise eine stärkere Fokussierung auf die Versorgungszeit bestehen mag.

Diese Interpretation wird unterstützt durch die Ergebnisse bei der Indikationsstellung zur Intubation im Schock beim nichtbewusstlosen Patienten. Hier kann eine Abweichung von Leitlinienempfehlungen durch den Notarzt wohl indiziert sein (Beispiel: fulminanter hämorrhagischer Schock und kurzer Transportweg in Zielklinik vs. protrahiert hypotone Kreislaufverhältnisse und langer Transportweg). Es zeigen sich für diese Indikation größere Unterschiede zwischen den Gruppen; insgesamt jedoch bei allen weniger Leitlinienadhärenz als bei der Intubation des Bewusstlosen. Die Option der individuellen Abwägung ist wesentliches Merkmal eines notarztgestützten Rettungsdienstsystems, welches nicht nur auf einer strengen Einhaltung von Algorithmen basiert. Abweichungen von Leitlinienempfehlungen können Zeichen eingeschränkter Leitlinienadhärenz oder aber – gerade bei den präklinisch erforderlichen, komplexen Indikationsstellungen – Ausdruck eines Bedarfs an hoher klinischer Erfahrung sein, die nicht vollständig durch Leitlinienvorgaben und Algorithmen substituiert werden kann.

### Zeitintervalle und Schockraumversorgung

Es zeigen sich statisch signifikante, absolut gesehen aber wenig relevante Unterschiede in der Versorgungszeit am Unfallort, was für eine sehr zügige Umsetzung der (häufigeren) präklinischen Interventionen in ANÄ spricht.

Kulla et al. konnten nachweisen, dass der Zeitpunkt der Durchführung einer invasiven Maßnahme (Präklinik vs. Schockraum) keinen Einfluss auf die Zeitspanne bis zum Ende der Schockraumversorgung (Prähospitalintervall plus Schockraumversorgung) hat [17]. Trotz liberaler Indikationen bei präklinischen Maßnahmen ist in der vorliegenden Arbeit die Schockraumdauer in ANÄ länger als in INN/CHIR und steht damit auf den ersten Blick im Widerspruch zur Arbeit von Kulla et al. Neben der unterschiedlichen Verletzungsschwere lässt sich dieses Phänomen aber auch dadurch erklären, dass die geringere präklinische Invasivität in INN und CHIR nur partiell im Rahmen der Schockraumversorgung kompensiert werden musste.

Durch die Unterschiede im Verletzungsmuster und in der Verletzungsschwere sowie die potenziellen Unterschiede in den Prozessabläufen der einzelnen Zielkliniken sind Schockraumzeit, Beatmungs‑, Intensiv- und Krankenhaustage jedoch nicht uneingeschränkt vergleichbar. Unklar bleibt, warum in der Gruppe ANÄ, mit der höchsten präklinischen Leitlinienadhärenz, noch 11 % der Patienten im Schockraum intubiert wurden. Möglicherweise ist dies durch innerklinische Prozesse bedingt (z. B. Intubation im Schockraum vs. Intubation im OP bei dringlicher Operationsindikation).

### Mortalität

Trotz etwas abweichender Versorgungsstrategien in den Fachgebieten besteht eine vergleichbare Krankenhausmortalität, die nicht durch die fachliche Zugehörigkeit des Hubschraubernotarztes beeinflusst wurde.

### Limitationen

Es handelt sich um eine retrospektive Datenanalyse mit den bekannten Limitationen dieses Studiendesigns. Vitalparameter und Interventionen werden für die notärztliche Versorgung nur zum Zeitpunkt Ankunft und Übergabe in elektronisch auswertbarer Form erfasst; eine potenzielle Dynamik während der präklinischen Versorgung sowie die individuelle Entscheidungsfindung beim einzelnen Patienten sind elektronisch derzeit nur bedingt abbildbar. Die Qualität der Daten wird durch die ärztliche Dokumentationsqualität im jeweiligen Register bestimmt. Bei einer zeitkritischen Versorgung eines Schwerverletzten kann es vorkommen, dass ein Teil der Dokumentation retrospektiv erfolgt und dadurch die Fehlerwahrscheinlichkeit erhöht ist.

Bei einer Nachforderung der Luftrettung können Interventionen bereits durch den bodengebundenen Notarzt durchgeführt worden sein und werden dann nur partiell durch den Hubschraubernotarzt beeinflusst. Der hohe Facharztanteil, die Dominanz des Fachgebietes Anästhesie und der hohe *Case load* an Schwerverletzten in der Luftrettung sind nicht repräsentativ für den gesamten deutschen Notarztdienst.

Einzelne Vitalparameter, Interventionen sowie die Versorgungs- und Schockraumzeit sind nur für einen Teil der Patienten verfügbar. Mutmaßlich gibt es Unterschiede in der Schockraumprozessqualität der beteiligten Kliniken, die einen Einfluss auf Schockraumzeit und -interventionen haben dürfte.

Präklinisch verstorbene Patienten sind im TR-DGU und in dieser Studie nicht erfasst. Ein Einfluss der Notarztqualifikation auf die präklinische Mortalität ist jedoch nicht auszuschließen.

## Fazit für die Praxis

- Leitlinienempfehlungen zur Intubation des Schwerverletzten werden durch Notärzte aus allen 3 Fachgebieten in hohem Maße umgesetzt.
- Es zeigen sich Unterschiede in der Indikationsstellung für einzelne Maßnahmen, insbesondere außerhalb des Leitlinienrahmens.
- Eine Annäherung bei der Intubationsindikation zwischen den Fachgebieten wurde über den Untersuchungszeitraum beobachtet und ist als Ausdruck des Durchdringungsgrades der S3-Leitlinie Polytrauma/Schwerverletzten-Behandlung zu verstehen.
- Der fachliche Hintergrund des Notarztes in der Luftrettung scheint – trotz unterschiedlicher Leitlinienadhärenz und Invasivität – keinen Einfluss auf die Krankenhausmortalität zu haben.
- Die notärztliche Interdisziplinarität im Luftrettungsdienst scheint bei einer insgesamt hohen Qualifikation keinen Nachteil darzustellen.
